# Aloes and Hemorrhoids

**Published:** 1901-11

**Authors:** John H. Grant


### CORRESPONDENCE.

### ALOES AND HEMORRHOIDS.

*Editor Buffalo Medical Journal:*

Sir—I do not know whether others in the medical profession have noticed the effects of laxative pills on patients in producing hemorrhoids, but it has been my experience that all laxatives containing aloes, or its active principal—aloin, are prime factors, in men more especially, of causing hemorrhoidal conditions. One of the physiological actions of aloes, or aloin is the determination of blood to the part, producing a local congestion, and if its use be persisted in a fine crop of external piles is the inevitable result.

As about seven-tenths of all laxative pills and tablets contain aloin, physicians should be on their guard in prescribing them for any extended use. JOHN H. GRANT, M. D.

893 Ellicott Square.

